# Enhancing Buoyant force learning through a visuo-haptic environment: a case study

**DOI:** 10.3389/frobt.2024.1276027

**Published:** 2024-07-12

**Authors:** Luis Neri, Julieta Noguez, David Escobar-Castillejos, Víctor Robledo-Rella, Rosa María Guadalupe García-Castelán, Andres Gonzalez-Nucamendi, Alejandra J. Magana, Bedrich Benes

**Affiliations:** ^1^ Tecnologico de Monterrey, School of Engineering and Science, Ciudad de Mexico, Mexico; ^2^ Facultad de Ingeniería, Universidad Panamericana, Ciudad de México, Mexico; ^3^ Department of Computer and Information Technology and School of Engineering Education, Purdue University, West Lafayette, IN, United States; ^4^ Department of Computer Science, Purdue University, West Lafayette, IN, United States

**Keywords:** Buoyant forces, haptic devices, visuo-haptic simulators, educational innovation, higher education, professional education

## Abstract

**Introduction:** This study aimed to develop, implement, and test a visuo-haptic simulator designed to explore the buoyancy phenomenon for freshman engineering students enrolled in physics courses. The primary goal was to enhance students’ understanding of physical concepts through an immersive learning tool.

**Methods:** The visuo-haptic simulator was created using the VIS-HAPT methodology, which provides high-quality visualization and reduces development time. A total of 182 undergraduate students were randomly assigned to either an experimental group that used the simulator or a control group that received an equivalent learning experience in terms of duration and content. Data were collected through pre- and post-tests and an exit-perception questionnaire.

**Results:** Data analysis revealed that the experimental group achieved higher learning gains than the control group (*p* = 0.079). Additionally, students in the experimental group expressed strong enthusiasm for the simulator, noting its positive impact on their understanding of physical concepts. The VIS-HAPT methodology also reduced the average development time compared to similar visuo-haptic simulators.

**Discussion:** The results demonstrate the efficacy of the buoyancy visuo-haptic simulator in improving students’ learning experiences and validate the utility of the VIS-HAPT method for creating immersive educational tools in physics.

## 1 Introduction

Human haptic perception involves the integration of kinesthetic and cutaneous feedback, which are the core functions of haptic devices. The sense of one’s body’s position and movement is known as kinesthetic feedback, whereas cutaneous feedback refers to the stimulation that mechanical receptors beneath the skin’s surface detect. Haptic devices gather information from a virtual environment and interact with the user via a joystick or a similar interface. Haptic devices operate in two primary modes: admittance and impedance. Admittance devices control the end-effector’s position by measuring the force applied by the user and transmitting it to the virtual environment. On the other hand, impedance devices resist the user’s movements to simulate the sensation of touching or manipulating virtual objects. In the context of impedance-based haptic devices, these systems collect data on the user’s perceived position and, based on the simulation’s calculations, exert a corresponding force on the user to mimic interaction with the virtual environment. This force feedback is crucial for creating a realistic sense of touch and resistance, enhancing the immersive experience (Adilkhanov et al., 2022).

Recent psychological science studies suggest that touch can easily create detailed, long-lasting memories, and haptic devices facilitate human interaction with virtual environments (Hutmacher and Kuhbandner, 2018). This finding can benefit training and education settings. Haptic technology has been introduced in diverse areas to promote expertise and learning, such as industry, navigation, e-commerce, gaming, the arts, medicine, physics, biology, and education (Saddik, 2007; Hamza-Lup and Goldbach, 2020). For example, pilots are often trained in flight simulators where mid-air haptic devices are installed to control the plane course (Girdler and Georgiou, 2020). In the medical field, haptic training environments have been used to perform diverse surgical procedures, such as suturing (Ricardez et al., 2018). Dental training with artificial teeth has also used haptic tools, and oral implant therapy has used haptic technology as a relevant part of dental practice (Escobar-Castillejos et al., 2016; Escobar-Castillejos et al., 2020a).

The use of haptic environments for educational purposes has increased notably within the last two decades, especially for teaching abstract or complex concepts. Kinesthetic feedback, which allows users to experience virtual objects’ weight, resistance, and movement, is often emphasized over tactile feedback, which involves sensations of texture, temperature, or vibration (Rietzler et al., 2018). Although some studies discuss devices combining both kinesthetic and tactile feedback (Pantelios et al., 2004), the kinesthetic aspect is crucial in educational contexts as it enables a deeper understanding of forces and movements within virtual environments (Kamińska et al., 2019). Haptic technology, by providing this immersive experience, has been shown to enhance human perception and learning (Fernández et al., 2016; Coe et al., 2021). This enhancement is further explained through the lens of embodied cognition theory (Wilson, 2002; Shapiro, 2019; Walsh and Magana, 2023).

Visuo-haptic simulators have been used in education to illustrate basic laws of physics (Hamza-Lup and Sopin, 2009; Hamza-Lup, 2019; Hamza-Lup and Kocadag, 2019; Qi et al., 2020; Walsh et al., 2021). Some authors are convinced that haptic devices are comparable and, at times, better than actual physical lab experiments because, in the latter, a limited number of combinations can be performed Neri et al. (2020). For example, one way to understand the concept of friction is usually by using a certain number of wooden blocks sliding on inclined planes. Nevertheless, the combination of physical wooden blocks with inclined surfaces is limited. If visuo-haptic devices are used for the same purpose, the coefficient of friction values can be changed over a wider and continuous range, and more cues can be shown (Hamza-Lup and Baird, 2012; Neri et al., 2018a; Neri et al., 2018b). In the field of electromagnetism, visuo-haptic simulators have also been developed to understand the nature of electric and magnetic forces that allow the understanding of field forces (Neri et al., 2015; Magana et al., 2017; Shaikh et al., 2017; Gao et al., 2020; Gao et al., 2022).

As technology advances, game engines have been used to develop educational software, which has helped educational institutions approach interactive learning and development. Game engines are inherently designed for dynamic and real-time interactions, making them an ideal platform for incorporating haptic technologies. They offer robust physics engines and support for various input and output devices, which are essential for creating realistic and responsive haptic experiences. In a study by Rüdel et al., they developed a plugin to incorporate 3 and 6 degrees of freedom (DOF) in Unreal Engine (Rüdel et al., 2018). This helped the authors develop a visuo-haptic environment for blind and short-sighted people by using the engine’s collision detection library. On the other hand, Kind et al. used the Unity game engine to develop an assembly validation environment, specifically for cockpit installation in a car (Kind et al., 2020). By using this approach, the authors state that game engines enable the modularity and adaptability of simulated tasks.

While the development of visuo-haptic simulators in educational environments has allowed the development and creation of innovative interactive applications, there are some important difficulties, such as 1) design of the learning experience to optimize the use of haptic devices, 2) inadequate visualization of the variables and elements of the simulator, 3) poor and unrealistic correspondence between the actual physical phenomenon and the experience in the visuo-haptic environment, 4) delay between the haptic feedback force and the corresponding digital images, 5) overreaction and/or limits of feedback forces, 6) lengthy delivery times in the development and implementation of visuo-haptic environments, and 7) redundant and duplication of efforts throughout the implementation processes.

In accordance with the description of Education 4.0 by Noguez et al. (2021), the current study continues earlier research into the most effective ways to develop visuo-haptic environments for teaching physical sciences at the college level. In that research, a methodology to design visuo-haptic scenarios, named VIS-HAPT, was proposed. This methodology improves the visualization and performance of the visuo-haptic simulator and saves important development time. Therefore, in this paper, a case study using the VIS-HAPT method is shown, illustrating step-by-step how a visuo-haptic environment was made using game engines to help students better understand the topic of buoyant forces in the teaching of physics. In this environment, students can experiment with the buoyant force acting on bodies of different sizes and densities that are submerged in different liquids. This topic was selected due to the difficulties reported in the literature for students to understand the nature of the buoyancy phenomenon and the various misconceptions students have about it (Young et al., 2011).

Therefore, to identify if it is possible to successfully apply the VIS-HAPT methodology to implement the buoyancy phenomenon as a case study, we posed the following research questions.

1. *What is the effect of using the buoyancy visuo-haptic simulator on students’ learning gains as compared to a more traditional approach?*
2. *What are the students’ perceptions using the buoyancy visuo-haptic simulator?*

This paper is structured as follows: Section 2 introduces the theoretical framework. Section 3 summarizes the VIS-HAPT methodology for the buoyancy visuo-haptic simulator, with Section 4 presenting a case study on its design and implementation. The benefits of the VIS-HAPT methodology, its impact on student learning, and student feedback on the simulator are discussed in Section 5. Finally, Section 6 highlights the key findings, and Section 7 concludes the paper.

## 2 Theoretical framework and related work

### 2.1 Embodied cognition

Embodied cognition is a theoretical framework positing that cognitive processes are deeply rooted in bodily experiences and actions. This encompasses physical practices such as experiential learning, embodied simulations, and physical movement activities (Keijzer, 2002; Anderson, 2003; König et al., 2018; Farina, 2021). The framework suggests that learning is not solely an intellectual endeavor but also involves physical interaction. According to Mayer’s multimedia learning framework, students frequently learn more effectively when they engage in hands-on or experiential activities that allow them to use their senses and interact physically with the subject matter (Mayer, 2009).

The concept of embodied design is derived from embodied cognition, emphasizing the role of bodily actions in shaping thought and ideas. In the context of learning, embodied design implies that students can leverage gestures, movements, and their natural environments as valuable learning resources, facilitating the development of meaningful ideas (Abrahamson and Lindgren, 2014).

Recent theoretical advancements, such as the Embedded Cognitive Load Theory, propose leveraging the benefits of embodied modes of interaction for learning without overburdening cognitive resources (Skulmowski et al., 2016). The embodied design offers a lens to explore abstractions, evaluate student reasoning, and bridge concepts across disciplines. It provides educators a robust tool for crafting student experiences, including lesson plans and activities.

Integrating both visual and tactile channels in the learning process can lead to more profound comprehension. Incorporating haptic devices in educational settings, which meld the sense of touch with visual simulations, can enhance students’ grasp of various physical concepts. Several studies have highlighted significant learning improvements in understanding challenging physics concepts among students using visuo-haptic simulators compared to their counterparts who did not (Neri et al., 2018a; Hamza-Lup and Goldbach, 2020).

Research by Morris et al. (2007) and Qi et al. (2021) also supports this, examining the impact of combining haptic devices with visual cues *versus* using haptic devices alone. These studies collectively indicate that haptic integration can bridge learning disparities in comprehending intricate physics concepts.

### 2.2 Students’ misconceptions in buoyancy

Buoyancy is a physical phenomenon commonly found in everyday life, for instance, when observing a ball floating in a pool, a balloon released into the air, ice cubes floating in a beverage, or ships navigating in water. In the context of real-world applications, buoyancy’s relevance extends to numerous industrial processes, such as mineral separation, water treatment, and various hydrostatics and hydrodynamics applications in sectors like chemical, oil, and shipping industries. Recognizing the importance of buoyancy in these areas underscores students’ need to understand and apply buoyancy principles accurately.

The full comprehension of buoyancy is difficult to grasp at different education levels, but there are efforts to achieve this goal. Studies conducted by Gao et al. (2020; 2022) analyzed the conceptual development of buoyancy in 8th-grade students in China. The authors established four sequential phases and discovered seven characteristics indicating intellectual improvement. The stages took students from a basic understanding of single concepts like density, mass, or volume to a point where they could combine many concepts related to buoyancy. For instance, they learned how to find and determine the direction of buoyant force, figure out the submerged volume and relative density, and use Archimedes’ principle to explain how objects float in liquids. Recognizing the diversity in student’s learning, Gao et al. supported the customization of education by using real-time data analysis to improve their model.

The research by Minogue and Borland is a different study that complements the understanding of buoyant forces in higher education (Minogue and Borland, 2016). A group of 40 undergraduate students majoring in education at NC State University, USA, were subjected to examining the effects of haptic feedback using a buoyant visuo-haptic simulator. Their study compared outcomes between a group that received bimodal feedback (visual + haptic) and a group that received only visual feedback. In a post-pretest analysis, the two groups had no statistically significant differences in learning gains. However, the authors found that the visual + haptic cohort was likelier to use “haptically grounded” words like pushing, mass, gravity, and buoyant force. This could suggest that participants who were exposed to both modalities developed a specific type of cognition involving language and touch, which may indicate a deeper conceptual understanding compared to the participants who only had visual exposure.

Consequently, before defining more complicated concepts, students must grasp fundamental physical principles and subsequently combine and integrate these principles. Finally, students must gather, assess, and incorporate the connections between all these elements into a logical mental framework. This comprehensive understanding of buoyancy is challenging due to various factors. Many students have deeply rooted misconceptions, which results in an incorrect interpretation of physical phenomena (Ammase et al., 2019). To fully understand scientific concepts, it is necessary to identify and correct any misconceptions students may have.

Due to misconceptions’ significant impact on students’ conceptual knowledge, it is crucial to identify and effectively resolve them. Several methods are available to discover student misconceptions, such as open-ended questions, diagnostic evaluations, concept mapping, prediction-observation-explanation exercises, and interviews targeting certain episodes or phenomena. These strategies facilitate the clarification of students’ comprehension of topics while also identifying and correcting any misunderstandings they may have (Köse, 2008).

Common misconceptions regarding buoyancy include.

1. Volume, weight, or mass alone determines if an object floats or sinks.
2. Object’s shape or orientation determines if it floats or sinks.
3. Buoyancy depends on the volume of the liquid surrounding the object.
4. Buoyant force depends only on the object’s density.
5. Buoyancy affects objects floating in a liquid but does not affect objects sinking in the fluid.

It is important to make students realize that the buoyant force depends on the density of the fluid surrounding the object and not on the density of the object. Therefore, the condition for an object to float is simply that the density of the liquid is greater than that of the object, regardless of its mass, volume, shape, or orientation. This is a key concept that instructors should work on with their students when studying buoyancy (Heron et al., 2003; Loverude et al., 2003; Ünal, 2008; Yin et al., 2014).

This study aimed to develop, test, and evaluate a visuo-haptic scenario about buoyancy to see how well it helped students understand related ideas like relative density, buoyant force, and net force.

### 2.3 Visuo-haptic simulations for learning

In previous research, Noguez et al. (2021) described the VIS-HAPT method for developing realistic visuo-haptic scenarios to help engineering students learn science and physics concepts. This methodology is based on the use of game engines for visuo-haptic learning simulators. The proposed methodology consisted of four stages that integrated different aspects and processes, leading to meaningful learning experiences for the students. Using the VIS-HAPT methodology, the authors reported a significant decrease of approximately 40% in the development and implementation times of visuo-haptic simulators compared with previous efforts. They also highlighted the enhanced quality of the designed visuo-haptic environments that resulted from applying this methodology. They also commented on students’ perceptions of the benefits of using visuo-haptic simulators to enhance their understanding of physics concepts. The students consulted in the study reported that they improved their understanding after using the proposed methodology. The authors concluded that the incorporation of haptic technologies in higher education settings would certainly foster better student performance in subsequent real-world environments related to Industry 4.0.

For educational purposes, visuo-haptic simulators have been used in education to illustrate difficult physics concepts Qi et al. (2020). The use of haptic technologies is stimulating for students and enables the creation of spaces for both e-learning and simulation environments. In physics, some students fail to fully grasp the concept of buoyancy because it requires knowledge about density, fluids, forces, mass, and gravity. Therefore, efforts in the field of technology-enhanced learning focus on how students can understand the relationship between objects immersed in fluids and the related physics (Young et al., 2011; Gao et al., 2022).

The simulator created by Dobashi et al. (2006) is an example of how to interact with buoyant forces. They developed a virtual canoe simulator where the participant can row a virtual canoe using an in-house haptic setup. Forces and torques can be felt while interacting with the virtual water, where the authors used fluids’ dynamic equations to compute and render the fluid forces acting on the virtual canoe. The simulator aims to let the participant realize the interaction with water while controlling the virtual canoe. The authors stated that their simulation could reproduce distinctive features of water flow forces and how these affect the forces acting on the pointer object.

In the work of Minogue and Borland (2016), the authors developed a haptically enhanced simulation for learning about buoyancy. Their application studied buoyancy to physical dimensions, volume, density, and the resulting sinking or floating behavior. The simulator explores density concepts and forces applied to a sunk object. The object’s size, the fluid’s density, and the object’s density can be controlled using a graphic user interface. They tested the simulator with 40 undergraduate students divided into two groups: 22 participants received visual and haptic feedback, while 18 received only visual feedback. They found that the participants who received haptic feedback made more frequent use of physics terms such as “push,” “force,” and “gravity” when they were asked to describe the activity. Consequently, the authors suggest that it is a marker for relation-based reasoning.

Another work is the one described by Qi et al. (2021). They developed a different visuo-haptic simulator where students could select different sizes of the interaction object and the amount of liquid that it will be submerged in. The simulator lets users add visual cues, such as interaction forces, to improve their understanding of the buoyancy concept. The simulator also used Novint Falcon haptic devices, and 60 undergraduate participants tested the environment. The authors performed a pre-test to identify if participants had prior buoyancy knowledge. The study found that participants with high prior knowledge understood the concept better when both visual and haptic cues were provided. On the other hand, participants with low prior knowledge improved their understanding by using only haptic sensations. The authors commented that the task could become confusing when individuals with low prior knowledge noticed the added visual cues. Therefore, participants decided to continue with haptic cues only.

## 3 VIS-HAPT methodology

Previous research introduced a comprehensive methodology named VIS-HAPT to design, implement, and evaluate learning experiences that implement visuo-haptic devices for engineering students (Noguez et al., 2021). This approach facilitated the creation of enhanced learning experiences and superior-quality visualizations. The VIS-HAPT methodology unfolds in four distinct stages.

1. *Design of learning experiences.*
2. *Technological model development.*
3. *Calibration and testing.*
4. *Application and field studies.*

**FIGURE 1:**
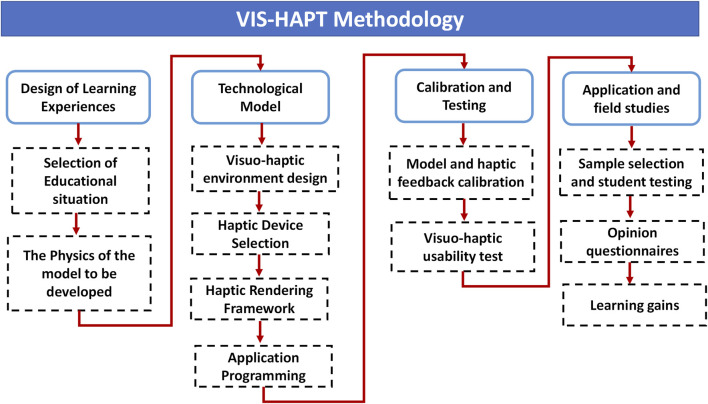
VIS-HAPT methodology.

These stages are illustrated in Figure 1. Notably, the methodology led to a significant reduction in time and effort across all stages. Below, a description of each of the main phases is presented.

### 3.1 Design of learning experiences

The design of virtual learning environments must consider an innovative learning experience that attracts students’ attention, where adding a sense of touch represents an advantage. In this phase, the objective is conceptualizing the visuo-haptic learning environment. The processes considered are: 1) The selection of educational settings, being crafted by first highlighting the main concepts of Physics and a careful simulator design of the physical phenomena that must be contextualized in a problem, and 2) Physics Modeling to model the characteristics and behavior of the bodies that will take place in the virtual learning environment.

### 3.2 Technological model

It considers the development of the application, the appropriate use of the screen space for interaction, and the integration of haptic devices into the simulation. This phase considers the following processes: 1) Visuo-haptic Environment Design to visualize the type of aid that would enhance students’ interactions, for example, a variety of views in the simulation, visual panels where interaction parameters will be displayed, and guiding lines to help students locate in the virtual space; 2) Haptic Device Selection, in which developers and physics experts should decide which haptic device is better suited for the interaction medium between the simulator and user; 3) Haptic Rendering Framework, which is the process by which the user can feel, touch, and manipulate virtual objects through a haptic interface; and 4) Application Programming which developers need to decide how the visuo-haptic environment can be best programmed.

### 3.3 Calibration and testing

In the methodology phase, developers and physics experts need to test the preliminary version of the visuo-haptic simulator. In this phase, two processes are carried out: 1) model and haptic feedback calibration, in which developers and physics experts test how the haptic device responds to the proposed physics model, and 2) visuo-haptic and usability tests, in which a group of users (usually students) are chosen to use the visuo-haptic environment and asked to do the tasks that the environment was designed to help your students do.

### 3.4 Application and field studies

In the methodology, the following aspects were proposed for applying the visuo-haptic environment in field studies: 1) choosing a sample that includes both control and experimental groups for student tests. It is important to note that when deciding how many experimental and control groups to use, the teaching resources given to each group must be equal, such as content, audiovisual materials, labs, class time, and so on. 2) Administration of opinion questionnaires that include questions related to the general usability of the system, its simplicity, and the accuracy between the system and the real world; and 3) measurement of student learning gains. In this final step, for the results to be meaningful, it is suggested during the process to have a sample as large as possible, both for the experimental and control groups, to have a professor teaching both the experimental and control groups to cancel as much as possible the “teacher effect”, and to administer the pre-test and post-test in similar and controlled conditions to both groups.

## 4 Buoyant forces case study

To solve the educational problems mentioned in Section 2.2, this study uses a case study to present how a visuo-haptic scenario can be used to help students understand how buoyant forces work on submerged objects. This scenario provides an immersive learning experience that helps students overcome common misconceptions and fully grasp the principles of buoyancy. We present the results obtained from the implementation by: 1) conducting control and experimental students’ groups, and 2) administering pre-test and post-test quizzes under controlled conditions.

### 4.1 Design of learning experiences

In the physics learning experience, abstract concepts may be difficult for some students to grasp. Therefore, physical phenomena must be chosen to contextualize them into attractive and challenging problems for the students. Generally, the design process for the learning experience begins with sketching the testing setup to visualize the phenomenon using the visuo-haptic simulator and the physical model equations to be implemented.

The main advantage of using a visuo-haptic simulator for buoyant forces instead of performing laboratory practices lies in the user’s ability to easily select various values for physical parameters such as the densities of the liquid and the immersed object, as well as the size or volume of the object. In simulations, users can observe the immediate effects of these choices on the flotation experiment. Achieving this level of flexibility in a physical setup would be quite challenging, as different liquids and objects with varying densities and sizes may not always be readily available. Moreover, altering the experimental setup each time a single variable is adjusted would be time-consuming.

Additionally, due to the haptic feedback provided by the simulator, students can directly *feel* the force required to submerge the object in the liquid, an aspect that would be difficult to replicate in a physical experiment. In a laboratory setting, a dynamometer could be used to estimate the buoyant force acting on the object, but students would not have a direct sensory experience of the force’s magnitude. In contrast, in visuo-haptic simulators, as the object is progressively immersed in the liquid, students can feel and observe how the strength of the buoyant force increases in real-time, and they can sense that once the object is fully submerged, the buoyant force remains constant.

On the other hand, while users could submerge the object in the liquid with their hands to perceive the force it exerts on them, registering its strength would be complex, and preventing the object from tilting would also pose a challenge. Furthermore, when the object’s density is greater than the liquid’s density, and the object sinks totally into the liquid, the proposed visuo-haptic simulator allows to push the object upwards from below and feel and register the corresponding force, but this would be very difficult or cumbersome to implement in lab experiments. Finally, it can be commented that the use of the relatively new haptic technology to perform experiments can spark in students their curiosity to inquire about these physics concepts.

#### 4.1.1 Educational scenario selection

**FIGURE 2:**
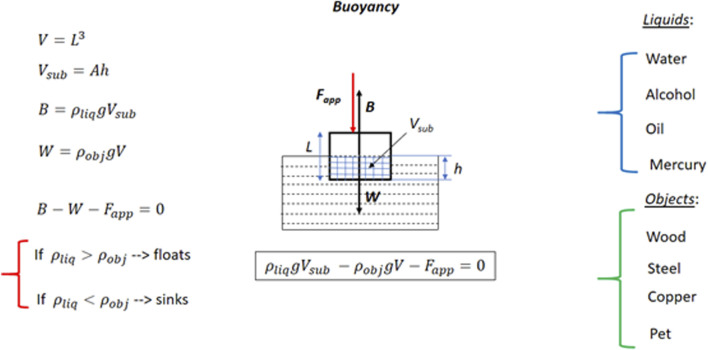
Example of the initial sketch design.

**FIGURE 3:**
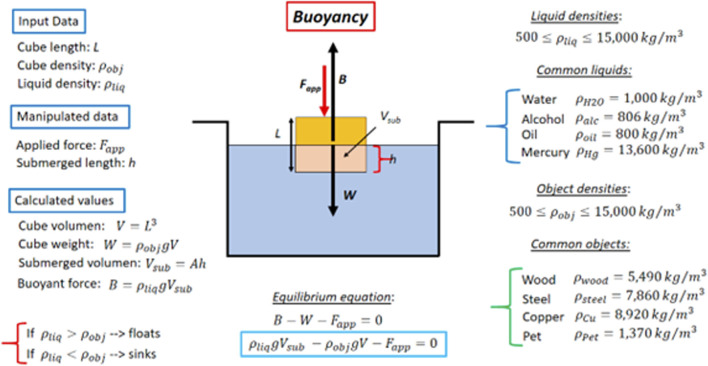
Detailed sketch design for buoyant visuo-haptic simulator.

According to the misconceptions that were identified, the main purpose of the visuo-haptic simulator is to let students learn what happens to the buoyant force when different parameters are modified, such as the density of liquids and object (denoted as *ρ*_*obj*_ and *ρ*_*liq*_, respectively) and the size of the object (represented by the length of a cube, *L*). These elements were incorporated into the design process from the initial and detailed sketches (see Figures 2, 3, respectively) to the final presentation of the simulator interface (see Figure 5).

Figure 2 shows the initial sketch for designing a visuo-haptic simulator for the buoyancy phenomenon, where the main physical parameters and equations are included.

• Gravity’s acceleration, *g*
• Object’s area, *A*
• Object’s volume, *V*
• Object’s density, *ρ*_*obj*_
• Object’s mass, *M* = *ρ*_*obj*_ *V*
• Object’s weight, *W* = *ρ*_*obj*_ *gV*
• Object’s submerged depth, *h*
• Object’s submerged volume, *V*_*sub*_ = *Ah*
• Liquid’s density, *ρ*_*liq*_
• Buoyant force, *B* = *ρ*_*liq*_ *gV*_*sub*_
• Magnitude of user’s applied force, *F*_*app*_
• Resultant force on object, *F*_*res*_

In the visuo-haptic simulator, all the physical quantities were computed using the International System.

#### 4.1.2 Physics of the model to be developed

The object’s shape was chosen as a cube of side *L* for simplicity. The corresponding detailed sketch is shown in Figure 3. By manipulating the haptic 3D joystick represented by a red sphere in the simulator, the user can apply an external force *F*_*app*_ at a desired point and direction within the simulator. The force can be applied downwards, upwards, or laterally on the cube by properly pushing the haptic sphere.

The physical variables that students are allowed to control in the visuo-haptic simulator are 1) the cube side length *L* ∈ [0, 10] cm, 2) the object’s density *ρ*_*obj*_ ∈ [500, 15000] kg/m^3^, and the liquid’s density *ρ*_*liq*_ ∈ [500, 15000] kg/m^3^. Additionally, for simplicity purposes, preset values for the object and liquid densities for some common substances can also be directly chosen on the simulator (copper, wood, PET, iron, water, oil, mercury, or alcohol).

Lastly, the forces, including the buoyant force *B*), are computed based on the user’s interactions and the selected physical parameters, as the Novint Falcon is an impedance haptic device. These calculated forces are then applied to the user via the haptic device. This method ensures that users receive direct, kinesthetic feedback corresponding to their actions within the virtual environment, enhancing the immersive learning experience.

### 4.2 Technological model

In the framework of the technological model, a recommendation is to use physics engines to reduce the modeling effort of both physics and programming (Escobar-Castillejos et al., 2020b). That is, it is possible to use the physics engines that are already available in the development frameworks. For example, the Nvidia PhysX engine can simulate the behavior of an object on the GPU and handle interactions between objects. Currently, game engines such as Unity and Unreal integrate physics engines to model physical behavior and can add visual appeal to the environment.

#### 4.2.1 Visuo-haptic environment design

**FIGURE 4:**
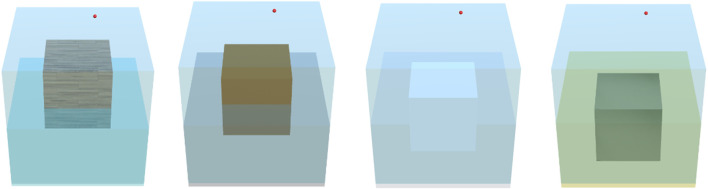
Examples of objects and liquids in the Buoyancy visuo-haptic simulator.

*Exploring shapes and objects* The next step in designing the visuo-haptic simulator involves exploring objects and shapes best suited for an appropriate and attractive visualization. Different textures and contrasts with neighboring objects and environments must be tested to provide users with an adequate perspective of the different components of the simulator. In Figure 4, several block textures and colors, as well as different liquids with different colors and various transparency degrees, are explored to provide the best visual experience to the user. Based on the detailed sketch, different object materials and liquids are incorporated. For example, in Figure 4, cubes of different materials such as wood, copper, PET, and iron are shown. Liquids are also represented with different colors to mimic water, alcohol, oil, and mercury. In this step, the physical properties of the cubes and liquids are also incorporated into the visuo-haptic simulator interface.

*Object manipulation tools* The bodies should accurately accelerate and be subject to forces like collisions, gravity, and other forces to ensure realistic physical behavior in the virtual environment. Unity’s Physics engine was used to simulate the fluid in the visuo-haptic environment. This engine considers in its calculations both solid and liquid bodies, but not individual particles. The simulator calculates and displays on the screen the following quantities: 1) the object’s mass *M*), weight *W*), and volume *V*), 2) the actual immersed object’s volume (*V*_*sub*_), 3) the submerged vertical depth of the object *h*), 4) the magnitude of the buoyant force exerted by the liquid on the immersed object *B*), 5) the magnitude of the applied (haptic) force on the object (*F*_*app*_), and 6) the magnitude of the resultant vertical force on the object (*F*_*res*_). The instantaneous values of these quantities are displayed on the screen. The vectors corresponding to the applied force, the buoyant force, the object’s weight, and a prompt with the instructions for using the simulator are also displayed on the screen.

The student’s interaction with the simulator is designed as follows: First, the student chooses the densities of the liquid, the object, and the cube’s length. The student can handle the haptic device by manipulating the haptic sphere (proxy point) into contact with any point at the top or bottom of the cube surface to push it up or down. For simplicity purposes, only applied vertical forces and, consequently, vertical displacements of the cube are allowed in the simulator. The forces applied sideways to the cube do not produce any lateral displacement. The simulator does not display any inclination or tilt of the cube, even when the external force is not applied exactly at the center of the upper or lower cube faces.

If the object’s density is higher than the density of the liquid, the object will sink in the simulator. In this case, an upward vertical force may be applied at the bottom of the cube to prevent it from sinking. If the object’s density is less than the density of the liquid, the object will float, with the immersed volume depending on these densities. In this case, a vertical upward or downward force can be applied to the cube to decrease further or increase the submerged volume. For all these experiments, the values for the immersed volume, the immersed vertical distance, the buoyant force magnitude, the applied force magnitude, and the total vertical force on the object are dynamically displayed in the simulator. In Figure 4, examples of the manipulation of different object materials and liquids are presented.

**FIGURE 5:**
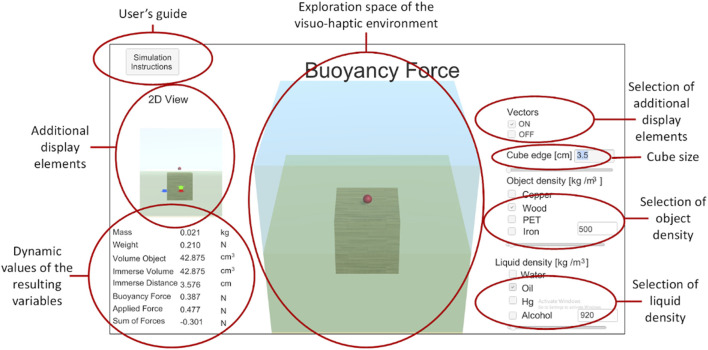
Final display of the buoyant visuo-haptic simulator interface.

*Interfaces* All previous elements are integrated into the final interface of the visuo-haptic simulator (Figure 5). A 2.5D visualization was developed for the visuo-haptic simulator, and its interface was designed to enhance its appeal to students and increase interactivity, enabling them to manipulate a proxy point to explore the depth dimension. A 2.5D visualization refers to a graphical representation that combines 2D and 3D elements, offering depth perception without the complexity of fully 3D environments. Students are required to locate the correct position of the proxy point to either push the object down from its top face or lift it from its bottom face. However, once the proxy point comes into contact with the object, it can only be moved vertically. Consequently, the object can only be submerged or lifted vertically. Any attempts to move it parallel to the liquid surface are restricted to prevent unintended lateral movements or tilting, which could divert students’ attention from the study’s primary objective: sinking or lifting the object in the liquid.

Within the graphical interface, users can activate or deactivate the visual representation of vectors. Moreover, they can manipulate the physical parameters directly related to the misconceptions held by students. These parameters include the densities of the object (*ρ*_*obj*_), the density of the liquid (*ρ*_*liq*_), and the side length of the cube *L*). Subsequently, the visuo-haptic simulator performs calculations and visualizes the aforementioned key physical quantities. Lastly, if required, users can open the user’s guide given to the students or the simulation instructions.

Additionally, a 2D free-body diagram (FBD) for the cube is displayed to explicitly illustrate the forces acting on it and guide the student’s understanding. Notably, in accordance with Newton’s third law of motion, if *F*_*a* *pp*_ represents the action force that the user applies, the haptic device generates it, and the user perceives the corresponding reaction force.

**FIGURE 6:**
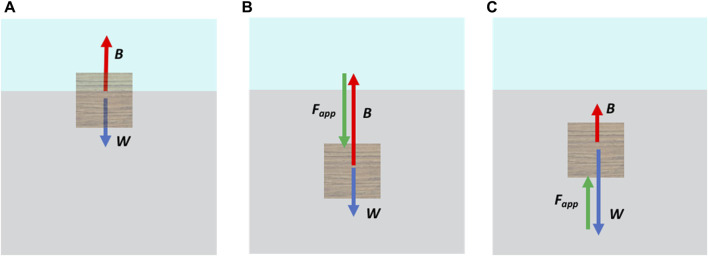
Free-body diagrams for a submerged object. **(A)** *ρ*_*obj*_ < *ρ*_*liq*_ without applied external force; **(B)** *ρ*_*obj*_ < *ρ*_*liq*_ with an applied downward external force *F*_*app*_, and **(C)** *ρ*_*obj*_ > *ρ*_*liq*_ with an applied upward external force *F*_*app*_.

Users can experience three cases in the visuo-haptic simulator corresponding to the force feedback the haptic device provides. From the object’s FBD (Figure 6) and taking into account the definitions given in Section 4.1.1, it can be observed that.

1. If *ρ*_*liq*_ > *ρ*_*obj*_, the object will float freely partially submerged in the liquid (Figure 6A). If no additional force is applied to the object with the proxy point, then *B* = *W*; therefore:

$$F_{app}=0.$$

2. If *ρ*_*liq*_ > *ρ*_*obj*_ and an additional force is applied downward on top of the object to submerge it further and maintain it in equilibrium, then *B* − *W* − *F*_*app*_ = 0 (Figure 6B); therefore:

$$F_{app}=B-W.$$

3. If *ρ*_liq_ < *ρ*_obj_, the object will sink. If the user wants to maintain it in equilibrium at a given depth, they have to apply an upward force on the object’s bottom, then *B* − *W* + *F*_app_ = 0 (Figure 6C); therefore:

$$F_{app}=W-B.$$

#### 4.2.2 Haptic device selection

As mentioned above, haptic devices are electromechanical tools used to recreate the sense of touch in virtual environments. Once the visual design and selection of the interaction are established, developers and physics experts should decide which haptic device is better suited for the interaction between a given physics simulator and the user. Haptic devices can be classified by the number of degrees of freedom and the degrees of force feedback provided by the device. Commercial haptic devices typically consider three, five, or six degrees of freedom.

**FIGURE 7:**
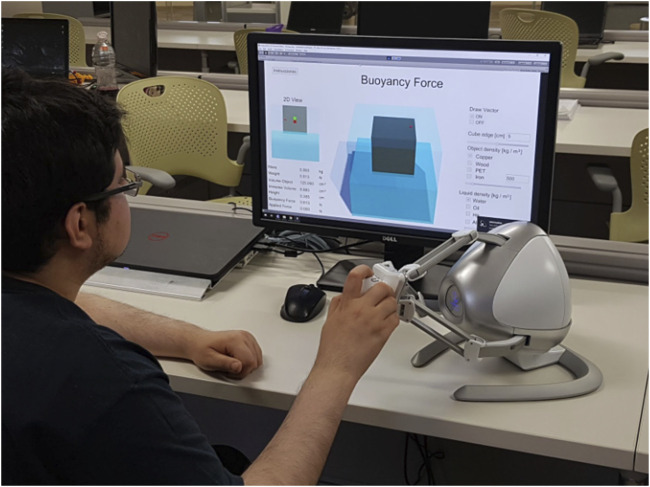
Interaction between the student and the bouyant visuo-haptic environment.

A haptic device with three degrees of freedom and three degrees of force feedback is adequate for the developed simulator, as its specifications align with the physical interactions within the buoyant forces simulation. Consequently, low-cost devices with three degrees of force feedback have been selected for this application, namely, the Novint Falcon haptic device. The device was also chosen as it may protect the user from potentially harmful forces by providing up to a maximum force feedback of 10 N, as further discussed in Section 4.3.1. The interaction between the student and the visuo-haptic simulator can be seen in Figure 7.

#### 4.2.3 Haptic rendering framework

Haptic rendering is the process by which the user can feel, touch, and manipulate virtual objects through a haptic interface (Saddik, 2007). In haptic-based simulators, the user interacts with the environment using a joystick. A renderer framework is needed to communicate applications with the haptic devices to enable haptic rendering.

The library implementation to correct connectivity between the application and the haptic device is a pitfall that must be worked out. Generally, haptic device manufacturers provide SDK to allow haptic representation in virtual environments. However, engine games do not have native functions that can easily admit them. The developers should make their own haptic rendering routines (Rüdel et al., 2018; Whitmire et al., 2018; Kind et al., 2020). Therefore, third-party solutions heavily depend on the CPU architecture and do not consider multiple devices’ operability. Moreover, developing a visuo-haptic solution usually takes a long time due to the complexity of graphic engines needed to deliver state-of-the-art visualizations.

To solve some of the mentioned difficulties, the HaDIU architecture was designed by Escobar-Castillejos et al. (Escobar-Castillejos et al., 2020b). It allows the successful development of multiple-device connectivity in game engines. At the same time, the application’s performance is not affected by multiple connections. One must have a functional blending of different environments, taking care of the connectivity of each device and optimizing the developing times of the visuo-haptic applications.

#### 4.2.4 Application programming

**FIGURE 8:**
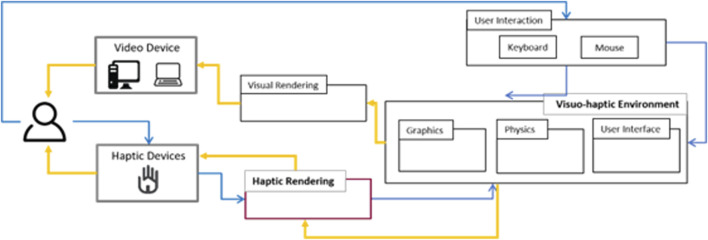
Pipeline for the development of the application programming for visuo-haptic environments.

Previous efforts focused on incorporating haptic devices in game engines can be found in Escobar-Castillejos et al. (2020b), where the authors added the connectivity of haptic devices (Novint Falcon, Omega, and Geomagic Touch) with Unity game engines. This is an example of how current approaches are considering the use of game engines to develop visuo-haptic simulators. As mentioned before, game engines are software applications that facilitate the implementation of graphics rendering, collision detection, and physics simulation, which allow the developer to focus on the game’s logic and interactions. Figure 8 shows the pipeline for developing the application programming for visuo-haptic environments proposed in this research.

### 4.3 Calibration and testing of the environment

In this phase, three developers and physics experts tested the preliminary version of the visuo-haptic simulator.

#### 4.3.1 Model and haptic feedback calibration

In the simulation, the haptic device operates in an impedance mode, where the proxy position is fetched at each frame. Upon contact with the virtual object, the interaction forces are calculated using the formulas defined in Sections 4.1.1 and Section 4.2.1. While Unity’s physics engine simulates the displacement of the surrounding fluid visually, all force calculations relevant to the user’s kinesthetic experience are derived directly from the specified physics formulas. Furthermore, the haptic response was calibrated to the user using a PID controller. This step became necessary because, during the initial stages of development, the simulation exhibited certain vibrations in its response to users. The range for the force magnitude delivered by the haptic device is 10 N, and the force calibration was facilitated by the Unity libraries and the PID controller. Consequently, the force the user feels is proportional to the feedback force (*F*_*app*_) the haptic device provides. Additionally, physics experts tested the simulator and validated the values calculated by the simulator, including the force feedback felt by the students. The reaction of the haptic device was tested according to the proposed Physics model. These tests were essential and crucial since they let the developers establish the limits in the simulation to avoid instabilities during simulation, for example, removing interaction variables and setting ranges or fixed values to some physical parameters.

#### 4.3.2 Visuo-haptic and usability tests

The calibration of the behavior of bodies within the visuo-haptic environment was based on the physical model described in Section 4.2.1. Moreover, a PID controller was programmed in Unity to manage the force feedback provided to the user, mitigating any abrupt fluctuations in force that could diminish the simulation’s realism.

In addition to calibrating the bodies’ behavior according to the physical model designed by the experts, usability tests were carried out with representative users who used the environment. These usability tests measure the ease of use of the different objects within the visuo-haptic environment. The users were asked to carry out the designed tasks. The interaction is registered, emphasizing the errors and difficulties the user encounters.

The usability metrics used successfully in this visuo-haptic environment were.

1. The accuracy in identifying the errors made by the test students and whether they were corrected with the appropriate data and procedures.
2. The time required to complete the activity.
3. The user memory allows for knowing how much students remember their interaction with the visuo-haptic simulator after some time without using the environment.

### 4.4 Application and field studies

Once the visuo-haptic environment was tested to ensure it worked with the physics laws shown in the models and with the right usability metrics, it was used in real classrooms to see if it could impact students’ learning gains.

#### 4.4.1 Sample selection and student testing

A quasi-experimental study starting in the August-December 2018 term was conducted with freshman undergraduate engineering students at Tecnologico de Monterrey, Mexico City Campus, to test the impact of the visuo-haptic simulator on students’ learning and motivation, as shown in Table 1. In 2019, a new educational model was started in our institution, and during 2020 and 2021, the confinement due to the COVID-19 pandemic prevented the use of in-site physical laboratories, so in the 2019–2021 period, the simulator was not tested. Other quasi-experimental studies started in February–June 2022 and the August–December 2022 term. In the latter, the authors conducted the study in similar conditions to the August–December 2018 term. In Table 1, the term and population of students participating in the study are presented.

**TABLE 1:** Students participating in the study.

Term	Group type	N
Aug-December 2018	Experimental	25
Aug-December 2018	Control	28
Feb-June 2022	Experimental	23
Feb-June 2022	Control	22
Aug-December 2022	Experimental	46
Aug-December 2022	Control	38
Total		182

Students of the experimental group had a 2-h session in the Cyber Learning Laboratory to work with the visuo-haptic simulator. Before beginning the hands-on exercises, the participants were requested to complete a written Pre-test about buoyancy concepts. This 25-min test was designed to assess their existing understanding of the subject. The Pre-test contained three multiple-choice conceptual questions and a problem where students had to calculate the immersed volume of a block inside a liquid. The pre-test and post-test questions addressed the misconceptions identified by students at our institution. The difficulty level for both the pre-test and post-test was consistent for both the experimental and control groups to ensure that any differences in learning between the two groups were attributed to using the visuo-haptic simulator. The questions were designed to help students realize two key concepts.

1. An object immersed in a fluid will float whenever the fluid density is greater than the object’s density (*ρ*_*liq*_ > *ρ*_*obj*_), regardless of the size or shape of the object.
2. The magnitude of the flotation force is equivalent to the weight of a volume of liquid equal to the immersed (or displaced) volume of the object within the liquid.

These concepts were integrated into both the pre-test and post-test designs and in the instructional guides provided to the students for their use with the visuo-haptic simulator.

Students of the experimental group were given about 15 min to become familiar with the simulator before using it. Before experimenting with the buoyancy simulator, experimental students were asked to give their consent verbally to participate in the study. Given the fact that the use of the visuo-haptic environment was not invasive, the rights of the experimental students were respected under the Declaration of Helsinki. After that, they were given an activity guide specially designed to develop different experiments focused on these misconceptions through several activities. The experiments included in the guide were.

• Placing a block in a liquid with higher density, allowing it to float freely to determine the submerged volume and buoyant force, and subsequently comparing these measurements with theoretical values.
• Partially submerging the block by pushing it into the liquid using the proxy point positioned on its top side in varying increments, without fully immersing it, and sensing the corresponding increase in applied force strength required for each increment. This involved comparing the values provided by the visuo-haptic simulator for buoyant force and submerged volume with those calculated using theoretical relationships.
• Completely submerging the block in the liquid to sense the applied force required for this action and noting that it remains constant regardless of the depth of the block within the liquid. This experiment allowed for the exploration of different values for object and liquid densities and object sizes.
• Placing a block in a liquid with lower density and observing how the object sinks, irrespective of the object’s size or the densities of the object and liquid.
• Lifting the submerged block and sensing the necessary applied force to maintain it at a specific depth. This involved performing relevant calculations and comparing the values obtained from the visuo-haptic simulator with those from the calculations.

#### 4.4.2 Opinion questionnaires

Experimental students worked with the simulator for about 60 min. At the end of the activity, they were asked to answer an exit questionnaire about their perception of using the simulator. During the Aug-December 2018 and Aug-December 2022 terms, a perception questionnaire on the use of the visuo-haptic simulator was applied, which is included in Table 2. A 5-step Likert scale was used for questions Q1–Q8, while questions Q9–Q11 were open-ended. The perception questionnaire was not applied during the February–June 2022 term.

**TABLE 2:** Perception questionnaire for the Buoyancy visuo-haptic simulator.

Id	Question	Scale
Q1	Please rate the difficulty level to select the simulation parameters	1 = Very difficult 2 = Difficult 3 = Neutral 4 = Easy 5 = Very easy
Q2	How motivated did you feel during the simulator session?	1 = Not motivated 2 = Partially motivated 3 = Indifferent 4 = Somehow motivated 5 = Very motivated
Q3	How attractive did you find the visualization of the 3D environment (2.5D)?	1 = Not attractive 2 = Partially attractive 3 = Indifferent 4 = Somehow attractive 5 = Very attractive
Q4	How realistic is the visual perception of objects in the 3D environment (2.5D)?	1 = Totally unrealistic 2 = Unrealistic 3 = Neutral 4 = Realistic 5 = Totally realistic
Q5	How realistic is the tactile perception of objects in the simulation?	1 = Totally unrealistic 2 = Unrealistic 3 = Neutral 4 = Realistic 5 = Totally realistic
Q6	Please rate the difficulty level when using the haptic device in the simulation	1 = Very difficult 2 = Difficult 3 = Neutral 4 = Easy 5 = Very easy
Q7	How useful do you consider the information the simulator offers to carry out the activities?	1 = Totally unuseful 2 = Unuseful 3 = Neutral 4 = Useful 5 = Totally useful
Q8	Do you consider that the use of haptic simulators supports you in learning the concepts discussed in class?	1 = Totally unuseful 2 = Unuseful 3 = Neutral 4 = Useful 5 = Totally useful
Q9	What suggestions do you have for the simulators to support better your learning process?	
Q10	What other experiments would you suggest to be implemented with haptic devices?	
Q11	General comments	

#### 4.4.3 Learning gains

To study the impact on learning of the implementation of the visuo-haptic simulator, a learning gain study was implemented during the Feb-June 2022 and Aug-December 2022 terms. This study was not considered for the Aug-December 2018 term. Experimental groups which used the visuo-haptic simulator and control groups were exposed to a comparable learning experience. As explained above, the same written pre-tests and post-tests were applied in class to both the experimental and control groups to compare the corresponding students’ learning gains. Table 3 includes the pre- and post-test questions, where the right answers for questions QA, QB, and QC are marked with an asterisk prefix.

**TABLE 3:** Pre/Post-test questions for the Buoyancy visuo-haptic simulator.

Id	Question	Options
QA	Two objects of the same volume *V*, with densities *ρ*_1_ > *ρ*_2_, are immersed in a fluid with density *ρ*_*L*_, with *ρ*_1_ > *ρ*_1_ > *ρ*_*L*_. If *B*_1_ and *B*_2_ are the buoyant forces on blocks 1 and 2, respectively, therefore	a) *B*_1_ > *B*_2_ *b) ***B***_**1**_ **= *B***_**2**_ c) *B*_1_ < *B*_2_ d) It is necessary to know the value of *V*
QB	Two objects of the same density *ρ*, with volumes *V*_1_ > *V*_2_, are immersed in a fluid with density *ρ*_*L*_, with *ρ* > *ρ*_*L*_. If *B*_1_ and *B*_2_ are the buoyant forces on blocks 1 and 2, respectively, therefore	* a) ***B***_**1**_ **> *B***_**2**_ b) *B*_1_ = *B*_2_ c) *B*_1_ < *B*_2_ d) It is necessary to know the value of *ρ*
QC	Two objects of the same density *ρ*, with volumes *V*_1_ > *V*_2_, are immersed in a fluid with density *ρ*_*L*_, with *ρ* < *ρ*_*L*_. If *B*_1_ and *B*_2_ are the buoyant forces on blocks 1 and 2, respectively, therefore	* a) ***B***_**1**_ **> *B***_**2**_ b) *B*_1_ = *B*_2_ c) *B*_1_ < *B*_2_ d) It is necessary to know the value of *ρ*
QD	A wooden cube block with sides of length *L* = 10 cm and density *ρ*_*m*_ = 800 kg/*m* ^3^ is placed in a container with water (*ρ*_*w*_ = 1000 kg/*m* ^3^). Find the submerged volume of the cube	The student is expected to perform a calculation and provide a response

The general objectives of the pre/post-test are that students realize that: 1) the buoyant force on a totally or partially immersed body in a liquid depends only on the immersed volume and on the density of the liquid, regardless of the density or the weight of the object; and 2) the immersed volume is equal to the block’s volume only if the block is totally submerged in the liquid. These are common misconceptions among our students, who think that the buoyant force depends on the body’s density and/or weight. The questions in these tests were designed to address these misconceptions from different perspectives.

The objective of question QA is to realize that the buoyant force on blocks with different densities completely submerged in a liquid depends on their volumes and on the density of the liquid, but not on their densities (that is, not on their weights either). Therefore, in this case, the buoyant force is the same for both blocks because they have the same volume, regardless of whether their densities are different: *ρ*_1_ > *ρ*_2_. The fact that the densities of the blocks are larger than those of the liquid (*ρ*_1_ > *ρ*_1_ > *ρ*_*L*_) only ensures that both blocks will completely sink in the liquid.

On the other hand, the objective of question QB is to realize that for objects with the same density *ρ* and totally immersed in the same liquid (with density *ρ*_*L*_), the buoyant force on the blocks only depends on their volumes. Therefore, in this case, the buoyant force on the largest block (block 1) is greater than that on the smallest block (block 2). The fact that the densities of the blocks are larger than those of the liquid (*ρ* > *ρ*_*L*_) also ensures that both blocks will completely sink in the liquid, as in question QA.

The objective of QC is to complement that of question QB, but in this case, the blocks are partially submerged in the liquid because their densities are smaller than that of the liquid (*ρ*_1_ = *ρ*_2_ < *ρ*_*L*_). The upward buoyant force balances the weight, and since *V*_1_ > *V*_2_, the weight of block 1 is larger than that of block 2, *W*_1_ > *W*_2_, therefore *B*_1_ > *B*_2_. This also implies that the immersed volume of block 1 is larger than that of block 2.

Finally, QD is a comprehensive problem where students must use their overall knowledge of the buoyant force to solve a quantitative equilibrium problem, like those found in typical textbooks. They must use the equilibrium equation to determine the submerged volume of the object, which is not the total volume of the block because it is only partially submerged in the liquid.

*Average learning gains* *G* For each section, the average pre-test <Pre> and average post-test <Post> were calculated for both experimental and control groups. The average absolute learning gain is calculated for each section according to the following relationship:

G=<Post>−<Pre>

*Relative learning gains* *G*_*rel*_

Relative learning gains, which represent the fraction of learning gain that a group of students obtained compared to the maximum possible absolute gain they can achieve, were also calculated for each section (Hake, 1998):

$$G_{\mathrm{rel}}=\frac{<Post>-<Pre>}{100-<Pre>}$$

The average pre-test and post-test values and the absolute and relative learning gains for the experimental and control groups are presented in Table 4 below.

**TABLE 4:** Learning gains for experimental and control groups.

Term	Type of group	*N*	*<Pre>*	*<Post>*	***G***	*G*_*rel*_
Feb-June 2022	Experimental	23	27.2	43.8	16.6	0.228
Feb-June 2022	Control	22	25.0	38.0	13.0	0.173
Aug-December 2022	Experimental	46	32.6	44.6	12.0	0.178
Aug-December 2022	Control	38	23.7	35.5	11.8	0.155

Experimental students, after performing the activities in the simulation, were also asked to answer a post-test equivalent in difficulty and length to the pre-test to determine whether they obtained any learning gains from using the visuo-haptic simulator. The pre- and post-tests were graded on a 0-100 point scale. In parallel, the control group students also answered the same pre-test as the one assigned to experimental students in their respective classes to maintain comparable interventions in both student samples. While experimental students worked with the visuo-haptic simulator, control students were given equivalent written texts and exercises from the textbook (e.g., Serway and Jewett, 2018) to ensure they also covered the buoyancy topics in their respective courses. In the next class session, control students were also asked to answer the same post-test assigned to experimental students to compare the learning gains of both groups. It is important to mention that the professor also briefly discussed buoyancy concepts with each group before the post-test was applied.

## 5 Results and analysis

### 5.1 Learning gains

In Table 4, the term, subject/section, type of group (experimental vs control), student population, average pre-test grade, average post-test grade, as well as average absolute and relative gains for each section are presented.

Table 4 shows that the absolute and relative gains are larger for the experimental groups compared to the control groups for both the Feb-June 2022 and Aug-December 2022 implementations. A *t*-test study was performed to determine if these gains are statistically significant, considering both Feb-June 2022 and Aug-December 2022 student samples. For this purpose, a cleansing procedure was performed. Student records in the data set with a 100 grade in both the pre-test and post-test were removed because these do not add information regarding the use of the visuo-haptic simulator. Outliers identified in box plot diagrams of relative learning gains for the experimental and control groups were also removed. This cleaning process yielded a sample of 62 experimental students and 49 control students.

The null hypothesis was:

$$H_{0}:\mu_{\exp}-\mu_{\mathrm{control}}=0$$

and the condition to reject it was:

$$H_{1}:\mu_{\exp}-\mu_{\mathrm{control}}>0$$

The results of the *t*-test for comparing means are presented in Table 5, where independent samples, as well as unequal and unknown variances, were assumed. To conduct the mean comparison test between independent populations, we utilized the Aspin-Welch *t*-test, which does not assume equal variances between populations. Additionally, we performed normality tests (Shapiro-Wilk, Kolmogorov-Smirnov, Anderson-Darling) in both groups, yielding *p*-values ranging from 0.01 to 0.1, indicating that the lack of normality is not significantly severe. The mean comparison test results are also presented in Table 5.

**TABLE 5:** Significance of learning gains.

Group	*N*	*G*_*rel*_	Mean standard error	Difference of means	*t*-test *H*_0_: *μ*_exp_ − *μ*_*control*_ = 0 *H*_1_: *μ*_exp_ − *μ*_*control*_ > 0	
Experimental	62	0.269	0.051	0.126	t	*p*-value
Control	49	0.143	0.072	0.126	1.42	0.079

It was found that the null hypothesis can be rejected with a *p*-value of 0.079. Although this value is higher than the common *p* = 0.05 threshold, there is a clear tendency for higher learning gains for the experimental group than for the control group. That is, to a significance level of 0.079, the use of the visuo-haptic simulator improves student learning of buoyancy concepts. Nevertheless, we are conscious that this possible learning gain is not completely satisfactory. This issue will be discussed in more detail in Section 6.

**FIGURE 9:**
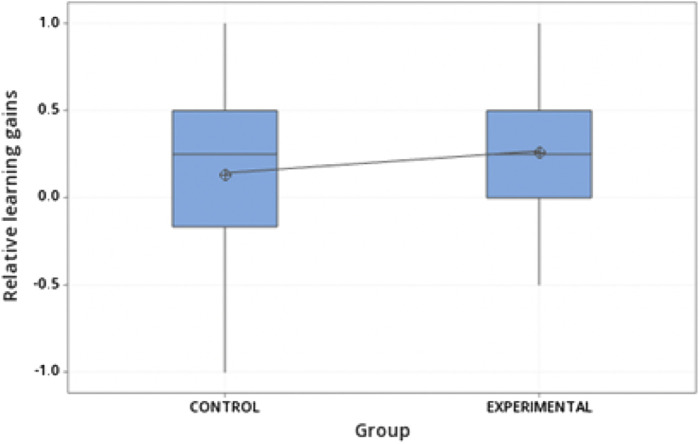
Box plots of relative gains for experimental and control groups.

The box plots in Figure 9 compare the relative learning gains for experimental and control groups. In these diagrams, it is seen that the average relative learning gain for the experimental group is not only larger than for the control group, but it is also more symmetrical and shows a smaller variability.

Also, separate tests were conducted to compare the means obtained in the pretest *versus* the post-test for both the experimental and control groups, based on the following hypotheses:

$$H_{0}:\mu_{\mathrm{Pos}}-\mu_{\mathrm{Pre}}=0$$

$$H_{1}:\mu_{\mathrm{Pos}}-\mu_{\mathrm{Pre}}>0$$

The result was that in the experimental group, the post-test mean was significantly greater than the pre-test mean (*p*-value = 0.007), while in the control group, the difference was less significant (*p*-value = 0.071). This suggests that using visuo-haptic simulators helps better understand concepts related to buoyancy.

### 5.2 Perception study

This section presents a study to determine students’ perceptions of using the buoyant force visuo-haptic simulator.

#### 5.2.1 Perception results

**FIGURE 10:**
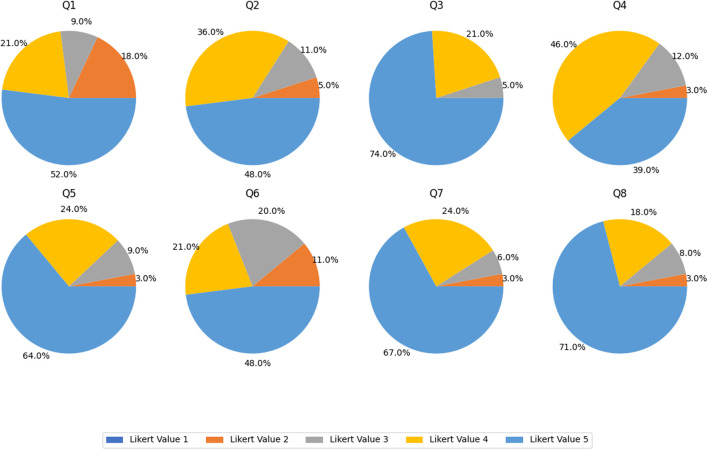
Pie charts representing the distribution of Likert scale responses for questions Q1-Q8 of the perception questionnaire.

During the Aug-December 2018 and Aug-December 2022 terms, the perception questionnaire presented in Table 2 was applied to engineering undergraduate students who used the Buoyancy visuo-haptic simulator. In Figures 10A–H, the corresponding pie charts of the total sample are also shown.

Figure 10 shows that overall, students had a very positive experience when testing the buoyancy visuo-haptic simulator features in both Aug-December 2018 and Aug-December 2022 terms. The results for both terms were very similar for all questions except for question Q1, which was higher in the Aug-December 2018 term. In general, students expressed an excellent opinion regarding the use of the simulator. The average values for all questions are higher than 4. The highest value corresponds to question Q3 (4.63), where students state that they appreciate the inclusion of a 3D (2.5D) visualization coupled with haptic feedback for the simulator. The students consider that the tactile perception of objects in the simulation was realistic, according to their everyday common experience. This is a relative consideration because each student responded based on their individual experience with the concept of force. The students felt the applied forces, which initially caught them by surprise. They expected to manipulate the object and observe values on the screen merely. However, when they started perceiving the applied forces, they found this aspect highly realistic. Queries Q7 (4.54) and Q8 (4.58) also received high perception ratings, indicating that students found the information provided by the simulator to be beneficial for performing the activities. They also commented that the visuo-haptic simulator helped them better understand the concepts studied in the classroom.

On the other hand, questions Q1 and Q6 obtained the lowest values, 4.06 and 4.08, respectively. They refer to the difficulty level when selecting the physical parameters in the simulator and when using the haptic device, respectively. Students commented on the difficulty of maintaining the block in a stable position, partially or completely submerged in the liquid, when applying a constant force, making it difficult to register the physical parameters because they changed quickly. It is interesting to notice that the Aug-December 2018 students did not perceive this problem as critical (Q1 = 4.43), while this issue was important for the Aug-December 2022 students (Q1 = 3.65).

Regarding the most common answers given to the open-ended questions (Q9 - Q11), students suggest instructors 1) give more support in class regarding buoyancy concepts, 2) give more detailed instruction on the use of the simulator before practicing with it, and 3) to provide a more clear and detailed Activities Guide for the haptic sessions. They also recommend the simulator provide more stable values because it changes “very quickly,” as commented above. The students also suggest implementing additional haptic experiments to include other physical forces such as spring, electric, magnetic, and hydrodynamic forces, among others. Finally, they expressed that they liked the haptic activity and qualified it as “very good,” “interesting,” and “attractive”.

### 5.3 Benefits of the buoyancy visuo-haptic simulator

Additionally to the promising students’ learning gains and the very good opinions from students, other benefits were obtained by using the VIS-HAPT methodology to develop the buoyancy visuo-haptic simulator. For the first part of the study, it was encouraging that development and implementation times remained on average for previous applications carried out with this methodology (Noguez et al., 2021). In this case, an earlier version of the buoyancy visuo-haptic simulator without methodology was not performed, so it was not possible to have a direct comparison.

However, as per our institution’s requirements, developers and researchers meticulously recorded a strict weekly log, where they recorded hours for the different stages of developing the visuo-haptic simulator using the VIS-HAPT methodology. For this case study, the times recorded for the different phases considered the following aspects.

*Design of learning experiences.* The time reported in this phase includes 1) the number of sessions and hours dedicated to the selection of the educational scenario, 2) the time necessary to design the contextualized physical phenomenon, 3) the time dedicated to the design of the visualization elements, and 4) the time invested in designing the physics model necessary to build the visuo-haptic environment. In total, this phase took 20 h.
*Definition of the technological model.* In this phase, we report 1) the time needed to prepare the visuo-haptic design, 2) the time spent on the definition sessions of the visuo-haptic device and the programming of its connections with the environment, 3) the time spent to enable the haptic rendering in the environment, and 4) the time it took to program the application. This phase was completed in 10 h.
*Calibration* and test. The time recorded for this phase includes 1) the programming of the PID controller, 2) the time to plan and run tests with average students, 3) the time needed to re-calibrate the models, and 4) the time spent planning, designing, and applying usability tests. This phase was finished in 12 h.
*Applications and field studies.* In this phase, the following times are considered: 1) the planning times registered for the implementation of the field studies with experimental and control groups; 2) the time necessary for the design of the activity guides delivered to the students during the learning experience; 3) the time needed to design and administer the pre-tests and post-tests; 4) the time needed to design and administer the opinion surveys; and 5) the time needed to analyze the data and results to measure learning achievement. This phase required 12 h.

The times reported for phases 1 and 4 were estimated from the hours recorded in our session records, considering the number of sessions carried out by our team working on the task (during the design of the learning experience), as well as through the different moments necessary to carry out the field studies of the visuo-haptic application with the students. It should be noted that one advantage of applying a methodology is that each process can be conducted in an orderly manner, considering the outcomes of each preceding phase. This optimization of development times facilitates the attainment of the expected results.

## 6 Discussion

Applying these teaching-learning hands-on techniques was especially motivating for this student generation, since coming back to on-site education after 2 years of pandemic lockdown has not been easy. The use of the VIS-HAPT methodology successfully designed a visuo-haptic simulator for buoyancy forces with a reduced average time-developing of only 55 h. This methodology also provided a high-quality simulator with appropriate 2.5D visualization incorporating visual aids such as buttons, sliders, and fields to select and display the values of the physical parameters in real-time, which helped students better perceive and understand the buoyancy phenomenon.

The presented visuo-haptic simulator incorporates and extends diverse capabilities that have proven useful in other buoyancy simulators. (Young et al., 2011; Minogue and Borland, 2016; Qi et al., 2020; Qi et al., 2021). The developed buoyancy visuo-haptic simulator offers a 2.5D visualization, enabling users to select physical parameters using a sliding bar effortlessly or by directly inputting values on the screen for densities and object size. Real-time display of relevant physical parameters and forces facilitates quantitative analyses, which are essential for undergraduate engineering students. This feature enables students to directly compare the magnitudes of calculated forces with the strength of feedback forces they experience while manipulating the haptic device. Additionally, it provides the option to display the FBDs of the submerged object, aiding students in better visualizing the forces involved in the buoyancy phenomenon.

Our quasi-experimental study indicates that the experimental group that used the visuo-haptic simulator obtained better learning gains than the control group, with a significance value of *p* = 0.079. Moreover, the average relative learning gain for the experimental group is also more symmetrical and shows smaller variability. Our findings suggest that the visuo-haptic scenarios’ embodied learning experience was more advantageous than the more conventional learning experience with less embodiment. In studying the derived learning gains, it is necessary to stress the importance of applying the pre-test and post-test instruments in similar conditions regarding difficulty level, time, and space to both the experimental and control groups. This may help to decrease any possible bias in the results. Although we recognize that the obtained *p*-value is above the commonly used 0.05 threshold and that the obtained learning gains are not completely satisfactory, it is noteworthy to see that the trend is in the expected direction, in which there is a tendency for students practicing with the buoyancy VHS to have larger learning gains than those who did not practice with it. In this way, as discussed in Section 2, buoyancy is a complex concept, and to grasp it fully, students might need more than just doing experiments in real labs or computers that simulate touch and movement. It has to be reinforced in the classroom with more detailed lectures, conceptual questions, and problem-solving. It is very likely that additional VHS practices in several sessions with more complete guides may be required for students to visualize better and internalize the related concepts in order to address the misconceptions.

Several studies have reported mild learning gains obtained by students working with buoyancy visuo-haptic simulators compared to control students not working with them or even with students working with only visual simulators without haptic feedback. In this way, Minogue and Borland (2016) did not discover significant differences in the learning gains for a buoyancy visuo-haptic simulator between undergraduate education majors who used visual and haptic (V + H) feedback and those who only used visual feedback (only-V). Nevertheless, the authors say that the simulator encouraged V + H students to use haptically-grounded physical terms (like mass, gravity, buoyant force, pushing, etc.) more often than only-V students. This finding may suggest that manipulating the visuo-haptic simulator helped the V + H students think more deeply. Young et al. (2011) reported similar findings for 4th and 6th grade students in elementary schools, highlighting that the visuo-haptic group did not perform significantly better than their visual-only group. Moreover, Qi et al. (2021) reported that the prior knowledge of undergraduate students on buoyancy and the visual cues added to their buoyancy simulator positively impacted students’ learning outcomes.

All of these results may suggest that the buoyancy phenomenon and the related concepts are difficult for students at different levels of education. As Gao et al. (2020; 2022) say, creating more detailed learning progressions should be important to help students better understand these concepts. Several incremental learning paths should be designed to guide students to internalize buoyancy concepts better. For this purpose, haptic practices alone might not be sufficient, so specific class lectures that include completing practical exercises should support and complement them.

The use of haptic devices to understand the nature of different physics forces besides the buoyant force (such as friction, mechanical, electromagnetic, elastic, etc.) has not always provided statistically significant learning gains when used by students of different academic levels. Some studies reported significant gains (Hamza-Lup and Baird, 2012; Neri et al., 2018b; Hamza-Lup and Goldbach, 2020), while others have not shown important improvement in student learning (Young et al., 2011; Neri et al., 2015; Minogue and Borland, 2016; Neri et al., 2018a; Neri et al., 2020; Qi et al., 2020; Qi et al., 2021). More research is needed in this field. Based on all of these studies, it seems that to get more out of using visuo-haptic simulators to help students learn about physical forces and performance, it is necessary to 1) combine them with problem-solving and class lectures, 2) give students more time to practice and interact with the simulators, 3) create more accurate guided simulator practices, and 4) plan more haptic activities to be spread out over the course of the semester.

Through the administration of perception questionnaires, first-year engineering students expressed positive experiences while using the visuo-haptic simulator. They stated that 1) they highly appreciated the inclusion of the depth dimension coupled with the haptic feedback for the simulator, 2) the tactile perception of objects in the simulation was highly realistic, 3) the information offered by the simulator to carry out the activities was useful, and 4) the visuo-haptic simulator helped them to understand better the concepts studied in the class. Moreover, they recommended implementing additional haptic experiments to include other physical forces, such as elastic, electromagnetic, and hydrodynamic forces. These results are in agreement with the findings of previous studies in which it became clear that students appreciated working with haptic technology coupled with visual simulators Hamza-Lup and Goldbach (2020); Hamza-Lup and Baird (2012); Neri et al. (2018a); Qi et al. (2020); Neri et al. (2015), Neri et al. (2020).

On the other hand, students in our sample suggested the following issues be considered in future haptic developments and implementations: 1) improving the stability of the system, in particular when the block had to be immersed in a fixed position inside the liquid; 2) providing more support in class regarding buoyancy concepts before practicing with the simulator; 3) giving more detailed instruction on the use of the simulator before practicing with it; and 4) providing a more detailed guide for the activities to carry out the haptic experiments.

Among the main limitations of this work, we can mention the following: First, since the goal of this study was to analyze how adding haptic feedback and a visual simulator affected student performance as a whole, it was not attempted to separate students into groups that only received visual feedback or haptic feedback to look at the effects of each modality on learning (Minogue and Borland, 2016; Qi et al., 2021). Secondly, as students have commented, the authors recognize that they must give students more time to become familiar with the visuo-haptic simulator before doing the practice. In this same direction, more time to practice with the simulator must be provided to students to fully take advantage of its capabilities, providing them with a more detailed experiment guide and allowing them extra time to explore it freely. Thirdly, the design of the simulator can be improved to 1) include other object shapes such as spheres and cones and 2) include a larger container with enough space to allow users to sink the object in the liquid to higher depths, recognizing that the buoyant force remains constant once the object is completely submerged in the liquid. This issue can be addressed in the current visuo-haptic simulator by selecting a smaller volume for the object. It is worth noting that during the 2018 implementation, the simulator experienced occasional vibrations in response to the user when the forces within it were substantial. In such cases, students were instructed to opt for smaller dimensions for the object and/or lower values for liquid/object density, effectively resolving the problem. The vibrations were rectified in the 2022 implementation, and we believe that they did not significantly impact the performance of the visuo-haptic simulator or, consequently, the student’s learning outcomes.

After the pandemic at our institution, we have observed a lack of concentration on behalf of the students, who get easily distracted by their electronic devices and chit-chat with their classmates. With the haptics activity, all the students were focused on what needed to be done, trying to master the joystick and even competing with the person next to them. We realized that haptic technology could get the students’ full attention. They all expressed interest, in agreement with the results reported by other authors (Young et al., 2011; Hamza-Lup and Baird, 2012; Neri et al., 2015; Neri et al., 2018b; Hamza-Lup and Goldbach, 2020; Neri et al., 2020). Young et al. ((Young et al., 2011)) also noted that since the visual environment and the sense of touch give life to abstract mathematical symbols, concepts like the magnitude of the buoyant force and its direction are simple to convey by a haptic device.

The haptic activity perfectly blends with the active learning philosophy since it provides a hands-on learning experience, students learn how to use other technologies, and at our institution, the activities are tailored to the student’s academic level and their interests. Moreover, the haptic activity contributes to the development of digital literacy, problem-solving, and critical thinking, which are among the most sought-after competencies by employers. Haptic scenarios provide more space for exploring the system than tapping into the motivational area. Even though buoyancy experiments can be done with ordinary materials found at home, the visuo-haptic simulator lets the user feel more like part of a learning game (Young et al., 2011).

According to embodied cognition principles, students in the visuo-haptic environment could both sense the applied force and observe changes in the FBDs simultaneously. This would not have been feasible in a traditional laboratory setting, where dynamic visual representations of forces acting on objects are not readily available. Abstract representations of forces, such as weight and buoyancy, can be challenging to comprehend. Real-world experience with how these forces behave is essential for accurately depicting them on paper. Cognitive processes at a higher level, including thought, language, and memory, rely on abstract representations (Keijzer, 2002; Anderson, 2003; König et al., 2018; Farina, 2021). After engaging in the visuo-haptic activities, students displayed increased confidence when tackling buoyancy problems, whether as homework or in class. Their free-body diagrams were clearer, and they correctly depicted acting forces, reducing anxiety during class.

Due to the relative usefulness of visuo-haptic activities at our institution, students vividly recall their experiences, even after they have completed the course. They discuss these experiences with their peers for an extended period of time. Students who did not have the opportunity to use the haptic device expressed disappointment to their professors for missing out. This suggests that students become emotionally engaged when working with the environment, aligning with the principles of embodied cognition.

## 7 Conclusion and future work

The VIS-HAPT methodology has proven to be suitable for designing appropriate visuo-haptic simulators to improve student performance in physics concepts, particularly the nature of buoyant forces. It allows for reducing important development times to design high-quality simulators with appropriate 3D visualization, which incorporates appropriate visual aids to select and display the values of the physical parameters in real time that help students better perceive and understand the underlying physical phenomenon.

Undergraduate engineering students who used the buoyancy visuo-haptic simulator created using the VIS-HAPT methodology obtained higher learning than those in the control group (with a significant difference of 0.079 *p*-value level). This suggests that adding touch to the simulator may help students learn more according to the embodied cognition framework. This result is encouraging and should motivate instructors to develop other experiments and use the resulting simulator in their courses to improve student understanding of difficult physics concepts. To obtain better results, it is important to consider 1) allowing students to become familiar with the simulator before implementing the haptic practice, 2) providing more detailed guides for the haptic practice, and 3) providing students with more time to practice with the visuo-haptic simulator and letting them also explore it freely according to their learning needs.

Including haptic activities in physics courses requires considerable effort and time in designing the visuo-haptic simulators and in the implementation logistics. Students also need more time to understand the gist of the buoyancy concept. Long-term learning requires constant supervision, feedback, and training to make the knowledge truly part of oneself. Overall, the haptic activity helped students to focus better on what they were doing, to motivate them, and to show that there are active ways to teach Physics in a more dynamic way than traditional lectures. The authors of this paper are truly committed to this academic journey to improve the quality of life and long-lasting learning.

As future work, our research group is dedicated to refining the VIS-HAPT methodology, which focuses on improving human-computer interaction (HCI) aspects. This focus is integral to fostering a more intuitive and engaging learning experience. We envision broadening the scope of the methodology to encompass authoring environments. This expansion will empower educators with the ability to customize and select the specific components that constitute the experiment in the visuo-haptic environments, tailoring the learning experience to best suit their educational objectives and the needs of their students.

Furthermore, the research group contemplates substantial advancements for the visuo-haptic simulator. One feature is to incorporate a variety of object geometries into the system, thus offering a more extensive and diverse collection of scenarios. A further feature under evaluation involves granting objects the ability to rotate when force is exerted at different points. This addition is projected to expand the simulator’s interactive capabilities and fortify learners’ understanding of how force applications can differently influence objects, depending on where they are executed. The research group also plans to test and implement higher-quality haptic interfaces, such as the Geomagic Touch (formerly Phantom Omni). Although these devices are more costly, incorporating them could address the stability issues reported by the students and provide a more seamless interaction within the visuo-haptic environment.

Finally, future studies will incorporate a focus on the gender and age demographics of the participants. Although the current study did not conduct any statistical analysis on these parameters, including them in future research could yield significant insights and facilitate the customization of learning experiences for different user groups.

## Data Availability

The original contributions presented in the study are included in the article/supplementary material, further inquiries can be directed to the corresponding author.
